# Bedside Fluorescence Microangiography for Frostbite Diagnosis in the Emergency Department

**DOI:** 10.5811/westjem.2022.8.55020

**Published:** 2022-10-23

**Authors:** Sarah M. Raleigh, Margot Samson, Rachel Nygaard, Fredrick Endorf, Joseph Walter, Thomas Masters

**Affiliations:** * Hennepin County Medical Center, Department of Emergency Medicine, Minneapolis, Minnesota; † Hennepin County Medical Center, Department of Surgery, Minneapolis, Minnesota; ‡ Hennepin County Medical Center, Department of Hyperbaric Medicine, Minneapolis, Minnesota

## Abstract

**Introduction:**

Frostbite leads to progressive ischemia eventually causing tissue necrosis if not quickly reversed. Patients with frostbite tend to present to the emergency department (ED) for assessment and treatment. Acute management includes rewarming, pain management, and (when indicated) thrombolytic therapy. Thrombolytic therapy in severe frostbite injury may decrease rates of amputation and improve patient outcomes. Fluorescence microangiography (FMA) has been used to distinguish between perfused and non-perfused tissue. The purpose of this study was to evaluate the potential role of FMA in the acute care of patients with frostbite, specifically its role as a tool to identify perfusion deficit following severe frostbite injury, and to explore its role in time to tissue plasminogen activator (tPA).

**Methods:**

This retrospective analysis included all patients from December 2020–March 2021 who received FMA in a single ED as part of their initial frostbite evaluation. In total, 42 patients presented to the ED with concern for frostbite and were evaluated using FMA.

**Results:**

Mean time from arrival in the ED to FMA was 46.3 minutes. Of the 42 patients, 14 had clinically significant perfusion deficits noted on FMA and received tPA. Mean time to tPA (measured from ED arrival to administration of tPA) for these patients was 117.4 minutes. This is significantly faster than average historical times at our institution of 240–300 minutes.

**Conclusion:**

Bedside FMA provides objective information regarding perfusion deficits and allows for faster decision-making and improved times to tPA. Fluorescence microangiography shows promise for quick and efficient evaluation of perfusion deficits in frostbite-injured patients. This could lead to faster tPA administration and potentially greater rates of tissue salvage after severe frostbite injury.

## INTRODUCTION

Frostbite occurs when small ice crystals form in tissue and perfusion is disrupted.^1^,^2^ The hands, feet, nose, and ears are the most commonly damaged areas of the body.^3^,^4^ If not quickly reversed, prolonged cold exposure leads to progressive ischemia eventually causing tissue necrosis.^5^ Frostbitten patients tend to present to the emergency department (ED) for assessment and initial treatment. A large percentage of these patients also suffer from psychiatric illness, substance use disorder, and other psychosocial issues that often complicate treatment.^6^,^7^

Acute frostbite management includes active rewarming, pain management, and (when indicated) thrombolytic therapy.^8^–^10^ Due to the environmental nature of this disease, much of the frostbite research has stemmed from relatively few institutions.^11^ These studies have suggested that thrombolytic therapy in severe frostbite injury may decrease rates of amputation and improve patient outcomes.^9^,^10^,^12^ One study found that each hour in delay of treatment with tissue plasminogen activator (tPA) resulted in a decrease of tissue salvage of 28.1%.^13^

Acute evaluation for frostbite in the ED is based on history and exam. However, physical exam alone is unreliable.^8^ Numerous imaging modalities have been used to assist with decision-making, including nuclear medicine bone scans and angiography. The time required to obtain these advanced imaging options can often be prolonged (if available at all) and can be resource and labor intensive.^14^,^15^ Furthermore, tPA is thought to be time sensitive, adding urgency to making the diagnosis of severe frostbite.^16^,^26^

Recently, the use of fluorescence microangiography (FMA) has been implemented to assess tissue perfusion.^17^–^19^ This test consists of injecting indocyanine green dye (ICG) through a peripheral intravenous line. The ICG subsequently binds to blood lipoproteins and travels to where there is blood flow. Using a near-infrared laser coupled with a camera, one can then visualize blood flow within 3–5 millimeters of the skin surface. The dye is hepatically metabolized and safe in patients with renal disease. The ICG also has a very short half-life (150–180 seconds) making it ideal for serial use and allows for repeat imaging.

Fluorescence microangiography has the benefit of being a bedside imaging option, providing the physician with real-time visualization of perfusion deficits. Historically, FMA has been used in flap assessment, peripheral arterial disease, and wound monitoring.^20^–^23^ The ability to assess the viability of tissue at the bedside allows for a rapid assessment from the emergency physician and to pursue definitive treatment. In addition, a prior study on patients in the subacute phase following severe frostbite injury showed microangiography to be similar to Tc99 bone scans when compared to final amputation level.^24^ To our knowledge, this is the first study in which bedside FMA was used in the ED to help determine the need for time-sensitive thrombolytics and assist in prognostication of future need for amputation. Our goal was to evaluate the potential role of FMA in the acute assessment of perfusion deficit of frostbite patients at the bedside. An exploratory outcome assessed the impact of this bedside assessment on reducing time to tPA.

## METHODS

This retrospective study included all patients from December 2020–March 2021 who received FMA with a SPY portable handheld imager (Stryker Corporation; Kalamazoo, MI) as part of their initial frostbite evaluation in a county ED. Per standard protocol, patients identified by prehospital or triage personnel to be at risk for frostbite were prioritized based on the general principle that there could be a threat to limb. Clinicians used FMA based on their clinical judgment. Patients had FMA if there was a clinical suspicion for significant frostbite, if their extremities were clinically rewarmed, and if they had no iodine allergy. The ICG dye is considered to have a very low incidence of clinically important side effects; however, per manufacturer guidelines, doses were titrated higher or lower for morbid obesity and children, respectively.

**Population Health Research Capsule**

What do we already know about this issue?

*Frostbite causes decreased perfusion to tissues. Fluorescence microangiography (FMA) is used to distinguish between perfused and non-perfused tissue*.

What was the research question?

*Can FMA be used in the acute setting to identify perfusion deficits following severe frostbite injury?*

What was the major finding of the study?

*Of the 42 patients who had FMA, 14 (33%) had clinically significant perfusion deficits and received tPA. Mean time to tPA was 117.4 minutes*.

How does this improve population health?

*Identifying severe frostbite rapidly in the acute setting may allow for faster time to tPA, potentially improving limb salvage in a frequently vulnerable population*.

Retrospective review of data collected included the following: age; gender; time from arrival to time of microangiography; time to Tc-99 triple-phase bone scans (when performed); time to thrombolytic administration; and amputations required within six months. Due to the challenging psychosocial factors implicated in frostbite care, each patient’s problem list was assessed for underlying comorbidities. Time to tPA for the patients treated for frostbite in the preceding two winter seasons was also analyzed to provide historical controls. Patients with no contraindications to thrombolytics, received tPA based on our institution’s frostbite treatment protocol that consists of a loading dose and a six-hour infusion. Our institution’s approach to frostbite has been discussed in previously published articles.^8^ This study received Institutional Review Board for Human Subjects Research approval at our institution.

## RESULTS

At a single site, 42 patients presented to the ED with concern for frostbite and were evaluated with FMA. Of those 42 patients, the mean age was 44.1 years, and the majority (78.5%) were male. Many patients had a diagnosis of substance use disorder (52.4%), mental health diagnoses (40.5%), or at least one medical comorbidity (40.5%). Of evaluated patients, nine received both bone scan and FMA (Table 1). Mean time from arrival in the ED to FMA was 46.3 minutes.

**Figure 1:**
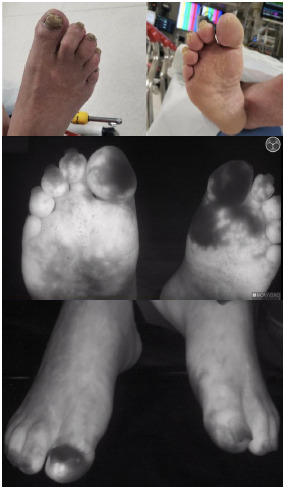
Use of bedside fluorescence microangiography in the emergency department for evaluation of a patient with possible frostbite. Lack of fluorescence (dark colored tissue in photos) indicates no fluorescein perfusion.

**Figure 2:**
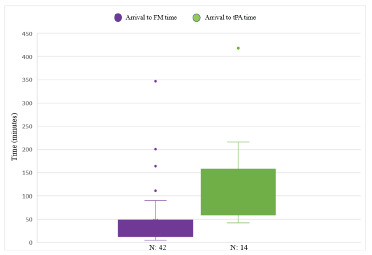
Time to fluorescence microangiography and time to tissue plasminogen activator from arrival in the emergency department. *tPA*, tissue plasminogen activator; *FM*, fluorescence microangiography.

Of the 42 patients assessed with FMA, 14 had clinically significant perfusion deficits (example in Figure 1). Fourteen patients received tPA. The mean time to tPA (measured from ED arrival to administration of tPA) for these patients was 117.4 minutes. This is compared to the two prior years’ average times of 348 minutes and 270 minutes. Time to FMA and time to tPA is shown in Figure 2. Outliers include patients who had other comorbid conditions or distracting injuries on arrival to the ED that were prioritized over the evaluation of their frostbite, and one patient who received a bone scan prior to FMA.

Of those who received tPA, five followed up in burn clinic and did not require any grafts or amputation afterwards, four had amputations, and five had no consistent follow-up (two died of other causes; two had intermittent follow-up in the ED, and one was lost to follow-up due to living out of state). Eight of the 42 patients eventually required amputations for their frostbite. Four of those had received tPA in our department. Of those who did not receive tPA, two were outside the window for tPA (>12 hours since frostbite injury), one received tPA at an outside hospital before being transferred (time to tPA unavailable), and one had slow perfusion on FMA and did not receive tPA.

Historically, bone scans have been used at our institution to evaluate for perfusion deficits after severe frostbite injury. Mean time to bone scan was 204 minutes in winter 2018–2019 and 318 minutes in winter 2019–2020. Time to tPA for these same winters was 348 minutes in 2018–2019 and 270 minutes in 2019–2020 (Table 2).

## DISCUSSION

Our first aim in this pilot study was to evaluate FMA as a tool to rapidly identify perfusion deficit following frostbite injury. Compared with other modalities, FMA is portable, accessible directly in the ED, and is relatively easy to use. With FMA it was possible to significantly assist in the diagnosis of severe frostbite injury when physical exam alone was not evident. The ability to use bedside FMA to obtain more rapid information regarding perfusion deficits means that the decision to administer thrombolytics in the appropriate patient can be made more quickly. Additionally, this may reduce the need to provide empiric thormbolytics.

At our institution, patients are often treated empirically for severe frostbite injury based on clinical exam by the burn team; this is used most often when there are delays in access to Tc99 scan due to high number of frostbite admissions. Thrombolytics are not a benign drug; prior studies on tPA in strokes have shown risks including symptomatic intracranial hemorrhage, major systemic hemorrhage, and angioedema in 6%, 2%, and 5% of patients, respectively.^25^ Recent studies on IV and intra-arterial tPA in frostbite have shown complication (rates between 2.3–10 % (compartment syndrome, bleeding requiring transfusion, and hematoma).^26^,^27^ Thus, judicious administration of thrombolytics is an important decision that should be made with appropriate clinical information.

One of our exploratory goals in this study was to determine whether FMA could improve time to thrombolytics in patients with severe frostbite in the acute setting. As noted above, faster time to thrombolytics can significantly improve tissue salvage rates.^13^,^28^ For this reason, any intervention that can improve time to thrombolytics could have major implications in limb salvage and outcome for patients with frostbite. In this retrospective study, mean time to FMA for the 42 patients involved was 46.3 minutes. This prompt assessment meant that the decision to give tPA could be made quickly. Time to tPA in this study was just under two hours (117.4 minutes), marking an improvement in our institution’s historical values. Additionally, this is an improvement from prior studies, with reported times of 6–6.9 hours.^13^,^24^

We believe that time to FMA (and therefore time to tPA) can be further improved. Fluorescence microangiography was a new device in our ED the year of this study; therefore, there was an inherent learning curve when it was first implemented. As physicians become more comfortable and familiar with the device, time to FMA, and time to tPA will likely improve.

## LIMITATIONS

This study does have several limitations. This was a small, single-site study that we conducted during one winter season. A larger sample size would increase the significance of findings. In some instances, confounding factors in medical care increased time to FMA. If a patient presented with frostbite but was unstable or required immediate resuscitation, those needs had to be addressed and the patient stabilized prior to evaluating the frostbite injury. Both experience identifying frostbite injury and familiarity with FMA may influence the utility of FMA. There is an expected “learning curve” for physicians regarding proficiency in appropriate application and interpretation. Thorough training of all emergency physicians with imaging review and feedback would be critical to ensure uniform evaluation of frostbite.

Additionally, we do not have data on discrepancies of perfusion assessment between the bedside assessment by the emergency physician and formal perfusion assessment by Tc99 bone scans. Therefore, we do not propose that FMA be used as a tool for exclusion from tPA, but rather to rapidly identify those with perfusion deficit for expedited tPA delivery. Clinical decision-making is difficult to assess in a retrospective study, but the general principle at our institution is to expedite tPA therapy to give patients the best chance of limb salvage following frostbite injury. When enacting FMA in the ED initially, the expectation was that patients would also have the standard bone scan prior to thrombolytic therapy. However, there were multiple instances where physicians observed frank digit ischemia on FMA and felt that delaying tPA therapy to obtain a bone scan posed an ethical issue, as prolonged tissue ischemia may cause further tissue loss. All patients undergo our usual frostbite thrombotic-risk screening protocol, as developed by our institution and discussed in previous publications.^8^,^10^,^13^,^16^,^24^

Finally, the patient population that traditionally suffers from frostbite includes a large percentage of individuals with mental health and substance abuse disorders. There was a significant loss to follow up at six months. However, this is in line with previous studies of a similar population.^28^

## CONCLUSION

Fluorescence microangiography shows promise in quickly and efficiently evaluating perfusion deficits in potential frostbite injured patients in the ED. This retrospective data suggests that FMA may lead to faster thrombolytic administration and, therefore, potentially greater rates of tissue salvage after severe frostbite. Future studies should focus on large sample sizes and determining whether decreased time to tPA improves long-term outcomes for severe frostbite injury.

## Figures and Tables

**Table 1:** Demographics of enrolled patients, time characteristics of diagnostic testing, and therapies.

Enrolled Patients (N = 42)	
Age (years): mean, SD, [range]	44.1, 16, [15–79]
Male, n (%)	33 (78.5)
Mental health diagnosis, n (%)	17 (40.5)
Substance use, n (%)	22 (52.4)
Homelessness, n (%)	12 (28.6)
Traumatic brain injury, n (%)	5 (11.9)
Medical comorbidities, n (%)	17 (40.5)
Arrival to fluorescence microangiography time, min (SD)	46.3 (61.4)
Those who received tPA, n (%)	14 (33.3)
Time to tPA, min (SD)	117.4 (95.7)
Amputations, n (%)	8 (19)
Those who underwent bone scans, n (%)	9 (21)
Time to bone scan, min (SD)	425.8 (765)

*N*, number; *SD*, standard deviation; *tPA*, tissue plasminogen activator.

**Table 2:** Historical values of time taken to treat with tPA.

	Minutes to bone scan (SD, N)	Minutes to tPA (SD, N)
Winter 2018–2019	204 (150, 56)	348 (150, 46)
Winter 2019–2020	318 (600, 21)	270 (162, 12)

*N*, number; *SD*, standard deviation; *tPA*, tissue plasminogen activator.
